# Optimal Time Period to Achieve Temperature Stabilisation After Total Contact Cast (TCC) Removal for Assessing Dermal Temperatures in Active Charcot Neuro‐Osteoarthropathy

**DOI:** 10.1002/jfa2.70059

**Published:** 2025-07-25

**Authors:** Justin Bradley, Mollie Rumble, Jennifer Wong, Ming Yii, Michelle R. Kaminski

**Affiliations:** ^1^ Department of Podiatry St Vincent's Hospital Melbourne Victoria Australia; ^2^ Department of City Futures City of Stonnington Council Victoria Australia; ^3^ Diabetes & Vascular Medicine Unit Monash Health Victoria Australia; ^4^ Department of Medicine School of Clinical Sciences at Monash Health Monash University Victoria Australia; ^5^ Department of Vascular and Transplant Surgery Monash Health Victoria Australia; ^6^ Department of Surgery School of Clinical Sciences at Monash Health Monash University Victoria Australia; ^7^ Department of Podiatry Monash Health Victoria Australia; ^8^ School of Primary and Allied Health Care Monash University Victoria Australia; ^9^ Discipline of Podiatry School of Allied Health Human Services and Sport La Trobe University Victoria Australia

**Keywords:** Charcot foot, Charcot neuro‐osteoarthropathy, diabetic foot, skin temperature, thermometry

## Abstract

**Background:**

Dermal temperature differentials between limbs are used to monitor disease progression and support safe withdrawal of immobilisation in Charcot neuro‐osteoarthropathy (CNO). Despite the wide clinical use of dermal thermometry, there is a lack of evidence on the optimal temperature stabilisation period after removal of immobilisation devices, such as total contact casts (TCCs). This study aimed to investigate the optimal time period to achieve temperature stabilisation post removal of TCC for assessing dermal temperatures in active CNO.

**Methods:**

Over a 2‐year period, this within‐subjects repeated measures study recruited 12 adults with active CNO treated with TCC from a metropolitan high‐risk foot service in Melbourne, Australia. Participants were excluded if they had bilateral CNO, an active foot ulcer, an inflammatory foot condition (e.g., gout), peripheral artery disease or major lower limb amputation. In a temperature‐controlled room, dermal temperatures were recorded using an infrared thermometer after removal of TCC and contralateral footwear. Temperatures were recorded at 10‐min intervals from baseline to 90 min at 10 anatomical locations on each foot. Paired samples *t*‐tests or Wilcoxon signed‐rank tests explored temperature stabilisation at each anatomical site across the 10 time points.

**Results:**

Mean age was 55.1 (SD, 8.9) years, 75.0% were male and 83.3% had type 2 diabetes. All participants had peripheral neuropathy and a large proportion had history of foot ulceration (75.0%). The average duration of CNO was 2.9 (SD, 1.7) months, with most classified as stage 1 (91.7%), affecting the tarsometatarsal joints (58.3%) and midtarsal joints (83.3%). Overall, dermal temperatures had stabilised by 40 min for the Charcot (casted) foot and contralateral (non‐casted) foot.

**Conclusions:**

This is the first study to explore the optimal time period to achieve temperature stabilisation when assessing dermal temperatures in active CNO. Forty minutes appears to be an appropriate resting time to reach thermal equilibrium. Although this approach may improve the accuracy of dermal thermometry, the time period may not always be feasible in clinical practice.

**Abbreviations**
BKA: below knee amputation
CI: confidence interval
CNO: Charcot neuro‐osteoarthropathy
HRFS: high‐risk foot service
IPJ: interphalangeal joint
IQR: interquartile range
IWGDF: International Working Group on the Diabetic Foot
MD: mean difference
MTPJs: metatarsophalangeal joints
SD: standard deviation
TCC: total contact cast

## 1 Background

Charcot neuro‐osteoarthropathy (CNO) is a serious limb‐threatening condition caused by an inflammatory process in individuals with peripheral polyneuropathy. This condition often leads to destruction of bones, joints and soft tissues of the foot and ankle [1, 2]. The presence of neuropathy can delay assessment and presentation to medical professionals during the active phase [1], resulting in frequent misdiagnoses (∼ 48% of cases) [3, 4]. Such delays can also lead to severe foot deformity, ulceration and amputation [4, 5]. Therefore, early diagnosis and management are critical for preventing the rapid progression towards irreversible foot deformity and its associated complications [6]. There are many reported aetiologies of CNO, but in modern western societies, diabetes mellitus has emerged as the leading cause [5]. The true prevalence remains largely unknown due to the often missed or delayed diagnosis during the active phase. However, population‐based studies have estimated the prevalence to range between 0.4% and 13% among patients with diabetes [5]. More recently, it has been estimated that 1.6 million people diagnosed with diabetes are living with CNO globally [1].

Active CNO typically presents with localised swelling, erythema and increased temperature (i.e., > 2°C compared to the contralateral, non‐affected foot) [4, 5]. Given the local inflammatory response during the active phase, temperature monitoring with infrared dermal thermometry is often used by clinicians to assist in diagnosing CNO, monitoring its progression and ensuring safe withdrawal of immobilisation through evaluation of temperature differentials [1, 5, 7]. There are a number of dermal temperature testing techniques and anatomical sites described in the literature [8, 9, 10, 11, 12], but the non‐touch technique has been found to be the most reliable [9]. Despite the wide clinical use of dermal thermometry, there is a lack of evidence on the optimal time period to achieve temperature stabilisation after removal of immobilisation devices, such as total contact casts (TCCs). It is emphasised in international guidelines [1] that a consistent approach to dermal temperature testing is important to ensure that repeated measures can be compared to monitor progress [1]. Although a 15‐min acclimatisation period is often utilised [10, 13, 14, 15, 16, 17], this is primarily based on expert opinion and realistic workflows in an outpatient clinic setting, rather than high‐quality evidence.

Since people living with CNO who use immobilisation devices often experience decreased confidence and self‐esteem, feelings of entrapment, reduced mobility, and loss of autonomy in their daily activities [18, 19], it is crucial to ensure that temperature differentials are reflective of active inflammation rather than the insulative nature of an enclosed cast. This is essential for accurately assessing clinical progress and informing effective management strategies. Therefore, this study aimed to investigate the optimal time period to achieve temperature stabilisation post removal of TCC for assessing dermal temperatures in active CNO.

## 2 Method

### 2.1 Study Design

This was a within‐subjects repeated measures study, where participants attended a single session for data collection. Data were collected over a 2‐year period.

### 2.2 Ethical Approval

This study was approved by the Monash Health Human Research and Ethics Committee (RES‐19‐0000‐794L), and all participants provided written informed consent prior to enrolment and data collection.

### 2.3 Participants

**FIGURE 1:**
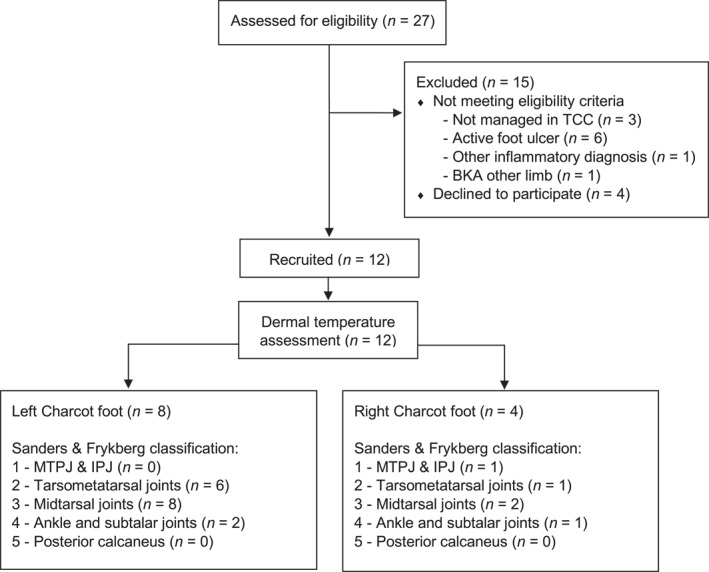
Diagram of participant flow through the study. BKA, below knee amputation; IPJ, interphalangeal joint; MTPJ, metatarsophalangeal joint; TCC, total contact cast.

All patients attending a metropolitan high‐risk foot service in Melbourne, Australia between June 2021 and March 2023 for management of CNO were assessed for eligibility. Participants were eligible if they were receiving TCC treatment for an active CNO (modified Eichenholtz stages 0–1) of the foot or ankle [20, 21, 22], were at least 18 years of age, were cognitively aware and had sufficient English language skills to provide informed consent and follow instructions during the study. Participants with bilateral CNO, an active foot ulcer, an inflammatory foot condition, such as gout or osteomyelitis, peripheral artery disease (defined as a systolic toe pressure less than 45 mmHg on either side) or those with a history of major lower limb amputation (defined as any resection proximal to the ankle [2]) were excluded. Figure 1 outlines the flow of participants through the study. Over the recruitment period, 27 prospective participants were screened for eligibility. Of these, four declined to participate due to additional travel and/or time commitments associated with study participation, whereas 11 were excluded based on the eligibility criteria. Overall, 12 participants were recruited and had their dermal temperatures assessed (Figure 1).

### 2.4 Procedure

Data collection initially consisted of an interview with the participant and a review of their medical records to obtain relevant data pertaining to sociodemographic characteristics, medical and foot history and clinical information (Tables 1 and 2). On any one day, two examiners (MR, JB or AW) were involved in the collection of data and performed the dermal temperature assessments. One examiner (JB) was a senior podiatrist with 16 years of clinical experience, whereas the other two examiners (MR and AW) were podiatrists with 5 and 15 years of clinical experience, respectively.

**TABLE 1:** Participant characteristics.

	Total (*N* = 12)
Age, years, *mean (SD)*	55 (8.9)
Male sex, *n* (%)	9 (75.0)
Ethnicity, *n* (%)	
Oceanian (Australia and New Zealand)	10 (83.3)
Southern and Eastern European	1 (8.3)
South‐East Asian	1 (8.3)
Identifies as Aboriginal and/or Torres Strait Islander person, *n* (%)	1 (8.3)
Smoking history, *n* (%)	
Never	9 (75.0)
Past	1 (8.3)
Current	2 (16.7)
Employment status, *n* (%)	
Full‐time	3 (25)
Not currently working due to foot health	3 (25)
Previously unemployed when CNO developed	6 (50)
Diabetes mellitus, *n* (%)	11 (91.7)
Type 1	1 (8.3)
Type 2	10 (83.3)
Duration, years, *mean (SD)* ^a^	21 (8.4)
HbA1c, %, *mean (SD)* ^a^	8.3 (1.9)
Previous foot complications, *n* (%)	
Ulceration	9 (75.0)
Amputation^b^	2 (16.7)
Peripheral neuropathy, *n* (%)	12 (100.0)
Retinopathy, *n* (%)	3 (25.0)
Nephropathy, *n* (%)	5 (41.7)
Comorbidities, *n* (%)	
Arthritis	4 (33.3)
Asthma	0
Back pain	3 (25.0)
Cancer	0
Chronic obstructive pulmonary disease	0
Congestive cardiac failure	1 (8.3)
Dementia	0
Dyslipidaemia	6 (50.0)
Hypertension	10 (83.3)
Depression	2 (16.7)
Mental health, other than depression	2 (16.7)
Osteoporosis	1 (8.3)

*Note:* Data are *n* (%), unless otherwise specified. Data may not add up to 100% due to rounding.

^a^ Maximum missing data were for HbA1c involving 8 participants overall. Missing data were for diabetes duration (*n* = 1).

^b^ The extent of previous amputation was of the hallux (*n* = 2).

**TABLE 2:** Charcot history and clinical presentation.

	Total (*N* = 12)
Charcot neuro‐osteoarthropathy, *n* (%)	
Right foot	4 (33.3)
Left foot	8 (66.7)
History of CNO, *n* (%)^a^	3 (25.0)
Precipitant to CNO, *n* (%)	
Minimal trauma^b^	3 (25.0)
Significant trauma^c^	2 (16.7)
Unknown/No obvious precipitant	7 (58.3)
Delayed presentation to HRFS, *n* (%)	9 (75.0)
CNO confirmation, *n* (%)	
Clinical assessment	2 (16.7)
X‐ray	7 (58.3)
Computed tomography	1 (8.3)
Magnetic resonance imaging	2 (16.7)
Stage of CNO, *n* (%)^d^	
Stage 0 (inflammation, normal radiographs)	1 (8.3)
Stage 1 (inflammation, fracture/subluxation)	11 (91.7)
Classification of CNO, *n* (%)^e^	
I (metatarsophalangeal and interphalangeal joints)	2 (16.7)
II (tarsometatarsal joints)	7 (58.3)
III (midtarsal joints)	10 (83.3)
IV (ankle and subtalar joints)	2 (16.7)
V (posterior calcaneus)	0
Deformity on examination, *n* (%)	
No visual deformity	4 (33.3)
Forefoot	0
Midfoot bony prominence/partial collapse	5 (41.7)
Rockerbottom foot	2 (16.7)
Ankle/Hindfoot	1 (8.3)
Systolic toe pressure, mmHg, *mean (SD)* ^f^	
Right	104.6 (21.0)
Left	107.7 (28.4)

*Note:* Data are *n* (%), unless otherwise specified.

Abbreviations: CNO, Charcot neuro‐osteoarthropathy; HRFS, high‐risk foot service.

^a^ Defined as a resolved Charcot foot (i.e., modified Eichenholtz classification stage 3 with long‐term offloading in place).

^b^ Defined as an event not expected to fracture healthy bone (e.g., trip).

^c^ Defined as anything greater than ‘minimal trauma.’

^d^ Staging as per the modified Eichenholtz classification.

^e^ Classification as per the Sanders and Frykberg classification.

^f^ Missing data were for systolic toe pressures (left, *n* = 3; right, *n* = 4)

#### 2.4.1 Neurovascular Assessment

Peripheral neuropathy was assessed using a Semmes‐Weinstein 5.07/10 g monofilament at the plantar hallux and plantar first and fifth metatarsophalangeal joints (MTPJs) of both feet. Aligning with the International Working Group on the Diabetic Foot (IWGDF) practical guideline [23], neuropathy was documented if two out of three answers were incorrect at any of the three sites on each foot. Arterial status was determined by evaluating pedal pulses (dorsalis pedis and posterior tibial), Doppler waveforms using a Hadeco ES100V3Bidop Doppler ultrasound and systolic toe pressures at the hallux using a Hadeco photoplethysmography probe and toe cuff. Peripheral artery disease was recorded when reaching stage 1 or above on the Wound, Ischaemia, foot Infection (WIfI) classification system (more specifically, a systolic toe pressure less than 45 mmHg on either side) [24, 25].

#### 2.4.2 Dermal Temperature Assessment

**FIGURE 2:**
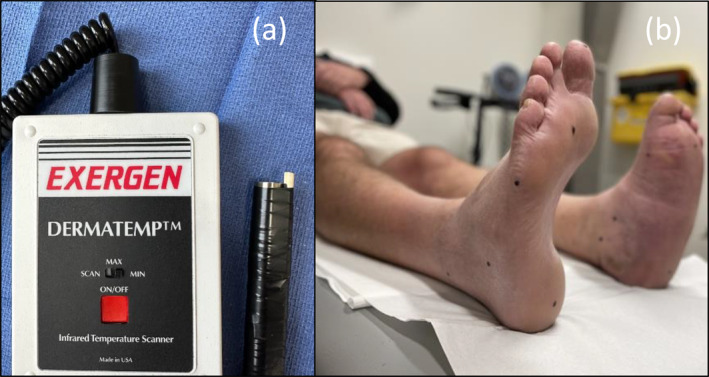
Dermal temperature assessment. (a) Exergen DermaTemp with 5 mm guide attachment and (b) marking of anatomical locations.

Dermal temperature measurements were conducted in a temperature‐controlled room, where the average room temperature was 22.5°C (standard deviation, SD, 0.4°C). At the start of each data collection session, the following were recorded: the ambient room temperature, the external air temperature (obtained from the Bureau of Meteorology observations website [26]) and the participant's body temperature, which was recorded using a Covidien Genius2 (Minneapolis, Minnesota) tympanic thermometer. To align with a previously published reliability study [9], we used the Exergen DermaTemp DT‐1001‐RS (Watertown, Massachusetts) infrared thermometer to perform a non‐touch technique (i.e., probe held 5 mm away from the skin's surface during measurement) when assessing dermal temperatures at 10 anatomical locations on both feet (Box 1). The anatomical sites were informed by previous research [9] and our existing high‐risk foot service Charcot assessment form. To ensure consistency in our measurements, all anatomical sites were marked with a felt tip pen and a 5 mm guide was attached to the infrared thermometer (Figure 2). In addition, the infrared thermometer was purchased specifically for use in this study to ensure calibration and accuracy as per factory standards.

**BOX 1 Anatomical testing sites.**

1

**Table:** 

Site	Anatomical location
1	Plantar heel
2	Lateral malleolus
3	Medial malleolus
4	Navicular tuberosity
5	Dorsal midfoot (base of the 3rd metatarsal)
6	Cuboid
7	Lateral 5th MTPJ
8	Plantar 3rd MTPJ
9	Medial 1st MTPJ
10	Plantar hallux

After removal of the participants' TCC and contralateral footwear, baseline temperatures were recorded within the first 2 min. The Exergen DermaTemp (Watertown, Massachusetts) infrared thermometer was set to the scan function, and using the attached guide, the thermometer was lightly applied to the marked anatomical testing site. This was to ensure that the thermometer was exactly 5 mm from the skin's surface during measurement [9]. The infrared thermometer was held for 3 s to allow for the temperature reading to stabilise. To reduce the risk of data entry errors, one examiner performed the dermal temperature assessment, whereas the other entered the temperature data directly into an online database (REDCap, Nashville, Tennessee, USA). Dermal temperatures were recorded at 10‐min intervals from baseline to 90 min at the 10 anatomical locations on each foot. A 10‐min testing interval was selected to provide adequate time to assess all 10 anatomical locations on both feet, while ensuring consistent time points were captured. The time required to measure and record dermal temperatures at the 10 anatomical sites on each foot was approximately 1.5–2 min. No specific temperature change threshold (e.g., a pre‐defined value in degrees Celsius) was used as a criterion for stabilisation. Instead, stabilisation was determined based on statistical significance, with the comparison of mean temperatures between consecutive time intervals (e.g., 10 vs. 20 min) assessed for significance at each anatomical site. Stabilisation was defined as the point at which the mean temperature differences were no longer statistically significant, providing an objective measure of thermal equilibrium. At the end of the data collection session, the participants' foot was washed and the TCC was reapplied as per the usual high‐risk foot service procedure.

### 2.5 Data Management

Statistical analysis was performed using the Statistical Package for the Social Sciences Version 23 (IBM Corporation, Somers, New York, USA), with the approach defined a priori. Descriptive statistics were initially calculated. Continuous data were expressed as mean (standard deviation, SD) or median (interquartile range, IQR). Dichotomous data were expressed as number (percentage). Normality of data for the distribution of the differences of the temperature measurements across the 10 time points were assessed using histograms, skewness and kurtosis values and Shapiro–Wilk statistical tests [27]. Vertical boxplots were used to assess for outliers. Of the ∼2400 temperature measurements recorded, 23 outliers were identified. Any data points that were more than three box‐lengths away (i.e., three interquartile ranges) from the upper and lower edge of their respective box were classified as extreme outliers and were addressed by replacement with the next non‐outlier value [27, 28]. Time point differences for temperature were expressed as mean differences (MDs) with 95% confidence intervals (CIs). Dependent on the normality of the data for the distribution of the differences, stabilisation of foot temperatures were explored using paired samples *t*‐tests or the Wilcoxon signed‐rank test. To address Type I statistical error from the large number of *t*‐tests performed (200 in total), the threshold for statistical significance was set a priori at *p* ≤ 0.01.

### 2.6 Sample Size

As this was an exploratory study, there were no previous studies in this specific area to inform the estimation of an appropriate sample size or statistical power. Consequently, we were unable to perform a formal sample size calculation. However, we aimed to include all eligible participants from our service to ensure that the maximum sample size was reached.

## 3 Results

We collected data on 12 adults with active CNO. Mean age was 55.1 (SD, 8.9) years, 75.0% were male and a large proportion identified as Oceanian (83.3%). The majority had type 2 diabetes (83.3%) with an average duration of 21.0 (SD, 8.4) years and glycated haemoglobin of 8.3% (SD, 1.9). All participants had peripheral neuropathy and a large proportion had history of foot ulceration (75.0%). The majority of participants had modified Eichenholtz stage 1 Charcot foot (91.7%) affecting the tarsometatarsal joints (58.3%) and midtarsal joints (83.3%). Trauma was a common Charcot precipitant (41.7%), although the precipitant was often unknown (58.3%). The average duration of CNO at the time of the dermal temperature assessment was 2.9 (SD, 1.7) months. Delayed presentation to the high‐risk foot service was evident in 75% of participants. A quarter of the sample (*n* = 3) had history of previous (i.e., resolved) Charcot foot. Participant characteristics and data pertaining to Charcot history and clinical presentation are shown in Tables 1 and 2.

**FIGURE 3:**
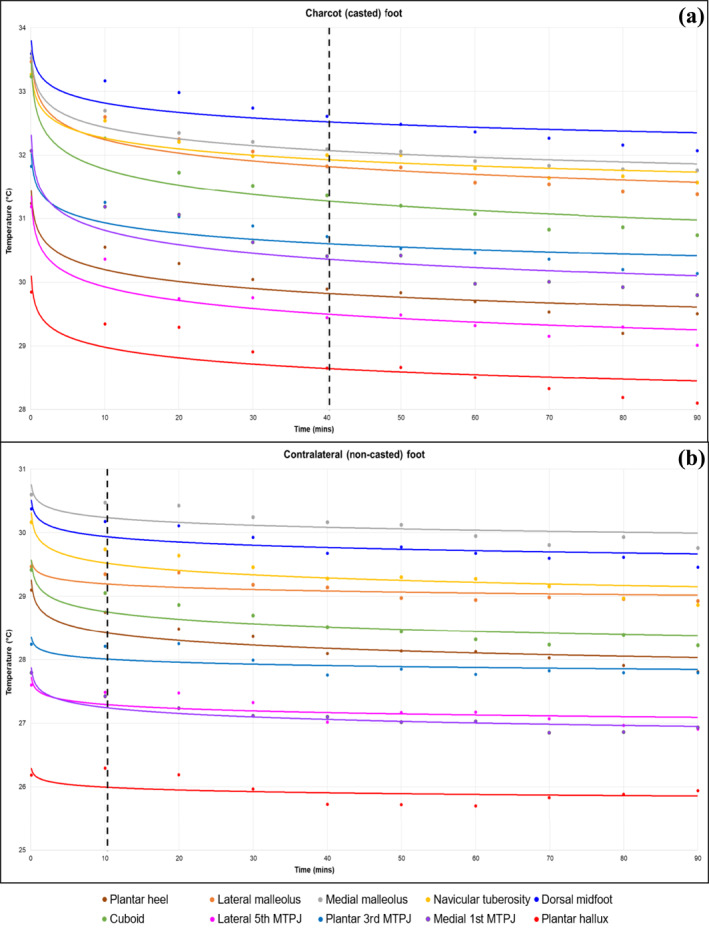
Mean dermal temperatures over time. (a) Charcot (casted) foot and (b) contralateral (non‐casted) foot. MTPJ, metatarsophalangeal joint. Dotted line represents the time point at which dermal temperatures had stabilised.

Table 3 presents comparisons of mean temperatures for the 10 anatomical testing sites across 10 time points for the Charcot (casted) foot and the contralateral (non‐casted) foot. Table S1 provides the mean (SD) dermal temperatures recorded. Overall, dermal temperatures at the 10 anatomical testing sites had stabilised by 40 min for the Charcot (casted) foot and contralateral (non‐casted) foot. For the Charcot (casted) foot, temperatures had stabilised by 40 min, with the exception of site 7 (lateral fifth MTPJ at 80 vs. 90 min) and site 9 (medial first MTPJ at 50 vs. 60 min), whereas temperatures had stabilised by 10 min for the contralateral (non‐casted) foot, with the exception of site 3 (medial malleolus at 50 vs. 60 min), site 5 (dorsal midfoot at 30 vs. 40 min) and site 7 (lateral fifth MTPJ at 30 vs. 40 min) (Figure 3). Significant differences in mean temperatures observed at these sites and time points are likely attributable to Type I error, as no clear trend was observed preceding these statistically significant findings.

**TABLE 3:** Comparison of mean temperatures (°C) at the 10 anatomical testing sites across 10 time points for the Charcot foot and contralateral foot—values are MD (95% CI) unless otherwise stated.

Site^d^	Baseline vs. 10 min	*p*‐value	10 vs. 20 min	*p‐*value	20 vs. 30 min	*p*‐value	30 vs. 40 min	*p*‐value	40 vs. 50 min	*p*‐value	50 vs. 60 min	*p*‐value	60 vs. 70 min	*p*‐value	70 vs. 80 min	*p*‐value	80 vs. 90 min	*p*‐value
Charcot foot (casted)																		
1	0.68 (0.05 to 1.32)	0.037	0.26 (0.01 to 0.51)	0.043	0.25 (−0.05 to 0.55)	0.095	0.15 (−0.06 to 0.36)	0.148	0.06 (−0.27 to 0.39)	0.703	0.14 (−0.11 to 0.12)	0.239	0.16 (−0.06 to 0.38)	0.143	0.34 (−0.08 to 0.76)	0.101	−0.31 (−0.88 to 0.26)^c^	0.305^e^
2	0.87 (0.44 to 1.30)	< 0.001^a^	0.35 (0.18 to 0.52)	< 0.001^a^	0.19 (−0.04 to 0.43)	0.099	0.23 (0.04 to 0.42)	0.020	0.02 (−0.14 to 0.18)	0.823	0.24 (−0.13 to 0.62)	0.183	0.02 (−0.24 to 0.29)	0.837	0.12 (−0.11 to 0.34)	0.284	0.04 (−0.30 to 0.38)	0.791
3	0.82 (0.48 to 1.17)	< 0.001^a^	0.35 (0.20 to 0.50)	< 0.001^a^	0.14 (0.04 to 0.25)	0.014	0.12 (−0.01 to 0.24)	0.062	0.03 (−0.10 to 0.17)	0.601	0.15 (0.03 to 0.27)^c^	0.019	0.08 (−0.10 to 0.25)	0.379	0.06 (−0.13 to 0.25)	0.518	0.02 (−0.16 to 0.19)	0.836
4	0.73 (0.39 to 1.06)	< 0.001^a^	0.33 (0.08 to 0.58)	0.013	0.23 (0.01 to 0.44)	0.045	−0.02 (−0.22 to 0.19)^c^	0.754^e^	0 (−0.11 to 0.11)^c^	1.000	0.21 (−0.06 to 0.47)	0.111	0.15 (−0.03 to 0.33)	0.098	−0.02 (−0.24 to 0.19)	0.798	0.10 (−0.05 to 0.25)	0.179
5	0.42 (0.14 to 0.71)	0.008^a^	0.18 (0 to 0.36)	0.048	0.24 (0.08 to 0.41)	0.008^a^	0.13 (0.02 to 0.24)	0.021	0.13 (−0.08 to 0.33)	0.202	0.12 (−0.04 to 0.27)	0.121	0.10 (−0.07 to 0.27)^c^	0.225	0.11 (−0.09 to 0.31)^c^	0.258	0.09 (0.01 to 0.17)	0.034
6	0.98 (0.53 to 1.42)	< 0.001^a^	0.53 (0.19 to 0.87)	0.003^a^ ^,^ ^e^	0.21 (0 to 0.42)	0.049	0.16 (−0.10 to 0.41)	0.199^e^	0.16 (−0.13 to 0.45)	0.258	0.13 (−0.19 to 0.46)	0.388	0.24 (−0.20 to 0.68)^c^	0.250	−0.04 (−0.26 to 0.17)	0.679	0.13 (−0.16 to 0.41)	0.356
7	0.83 (0.20 to 1.45)	0.014	0.62 (0.17 to 1.06)^c^	0.011	−0.02 (−0.27 to 0.24)	0.889	0.32 (−0.05 to 0.68)	0.085	−0.04 (−0.24 to 0.16)	0.658	0.17 (−0.11 to 0.44)	0.212	0.17 (0.01 to 0.33)	0.030^e^	−0.14 (−0.24 to −0.04)^c^	0.012	0.28 (0.18 to 0.38)	< 0.001^a^ ^,^ ^b^
8	0.58 (0.21 to 0.94)	0.005^a^	0.22 (0.08 to 0.37)	0.005^a^	0.14 (−0.07 to 0.35)	0.160	0.17 (−0.13 to 0.46)	0.239	0.19 (−0.11 to 0.49)	0.190	0.07 (−0.08 to 0.22)^c^	0.347	0.10 (−0.07 to 0.27)	0.231	0.17 (−0.06 to 0.39)	0.130	0.06 (−0.23 to 0.35)	0.668
9	0.88 (0.53 to 1.23)	< 0.001^a^	0.13 (−0.15 to 0.40)	0.339	0.43 (0.10 to 0.76)	0.014	0.22 (−0.11 to 0.55)	0.177	−0.01 (−0.29 to 0.27)	0.949	0.44 (0.20 to 0.68)	0.002^a^ ^,^ ^b^	−0.03 (−0.27 to 0.20)	0.761	0.09 (−0.09 to 0.28)	0.298	0.12 (−0.19 to 0.44)^c^	0.398
10	0.50 (−0.09 to 1.09)	0.087	0.05 (−0.19 to 0.30)	0.625	0.38 (−0.06 to 0.82)	0.082	0.25 (−0.16 to 0.67)	0.201	−0.01 (−0.36 to 0.34)	0.955	0.16 (−0.14 to 0.47)	0.256	0.17 (−0.05 to 0.40)^c^	0.118	0.14 (−0.11 to 0.38)	0.242	0.09 (−0.18 to 0.36)	0.464

Site^d^	Baseline vs. 10 min	*p*‐value	10 vs. 20 min	*p‐*value	20 vs. 30 min	*p*‐value	30 vs. 40 min	*p*‐value	40 vs. 50 min	*p*‐value	50 vs. 60 min	*p*‐value	60 vs. 70 min	*p*‐value	70 vs. 80 min	*p*‐value	80 vs. 90 min	*p*‐value
Contralateral foot (non‐casted)																		
1	0.35 (−0.06 to 0.76)	0.087	0.27 (0.06 to 0.48)	0.017	0.12 (−0.15 to 0.38)	0.359	0.27 (−0.18 to 0.73)^c^	0.207	−0.04 (−0.30 to −0.21)	0.724	0.01 (−0.33 to 0.34)	0.957	0.10 (0 to 0.20)	0.060	0.12 (−0.05 to 0.28)	0.147	0.10 (−0.16 to 0.36)	0.422
2	0.12 (−0.22 to 0.45)	0.464	−0.03 (−0.27 to 0.22)	0.828	0.19 (0.02 to 0.36)	0.032	0.04 (−0.13 to 0.21)	0.596	0.17 (−0.07 to 0.40)	0.145	0.03 (−0.26 to 0.32)	0.806	−0.04 (−0.28 to 0.20)	0.711	0.02 (−0.15 to 0.20)	0.757	0.03 (−0.19 to 0.25)	0.744
3	0.13 (−0.14 to 0.39)	0.327	0.05 (−0.09 to 0.19)	0.197^e^	0.18 (0.02 to 0.35)	0.030	0.07 (−0.10 to 0.25)	0.362	0.04 (−0.17 to 0.25)	0.667	0.18 (0.06 to 0.29)^c^	0.004^a^ ^,^ ^b^ ^,^ ^e^	0.14 (−0.16 to 0.45)	0.330	−0.12 (−0.41 to 0.16)	0.348	0.17 (0.02 to 0.33)^c^	0.033
4	0.42 (0.10 to 0.75)	0.016	0.10 (−0.11 to 0.31)	0.317	0.18 (0 to 0.36)	0.046	0.18 (−0.01 to 0.36)	0.063	−0.02 (−0.19 to 0.15)	0.832	0.03 (−0.21 to 0.26)	0.821	0.12 (−0.02 to 0.25)	0.084	0.19 (0.01 to 0.38)	0.043	0.10 (−0.05 to 0.25)^c^	0.172
5	0.20 (−0.05 to 0.45)	0.109	0.07 (−0.10 to 0.23)	0.394	0.18 (−0.11 to 0.48)	0.199	0.25 (0.11 to 0.39)	0.002^a^ ^,^ ^b^	−0.10 (−0.28 to 0.08)	0.246	0.10 (−0.15 to 0.35)	0.389	0.08 (−0.08 to 0.23)	0.298	−0.02 (−0.24 to 0.21)	0.686^e^	0.16 (−0.10 to 0.42)	0.222^e^
6	0.37 (0.05 to 0.68)	0.027	0.18 (−0.04 to 0.41)	0.100	0.17 (−0.05 to 0.38)	0.117	0.18 (−0.08 to 0.44)	0.136^e^	0.08 (−0.20 to 0.35)	0.561	0.13 (−0.11 to 0.36)	0.260	0.08 (−0.11 to 0.28)^c^	0.367	−0.15 (−0.36 to 0.06)	0.136	0.16 (−0.02 to 0.33)	0.074
7	0.12 (−0.38 to 0.61)	0.307^e^	0.01 (−0.32 to 0.34)	0.957	0.15 (−0.18 to 0.48)	0.337	0.31 (0.07 to 0.55)^c^	0.005^a^ ^,^ ^b^ ^,^ ^e^	−0.15 (−0.49 to 0.19)	0.352	−0.01 (−0.21 to 0.19)	0.929	0.11 (−0.10 to 0.32)	0.278	0.10 (−0.06 to 0.26)	0.209	0.06 (−0.05 to 0.17)	0.281
8	0.03 (−0.38 to 0.45)	0.864	−0.04 (−0.29 to 0.21)	0.838^e^	0.26 (−0.16 to 0.67)	0.198	0.23 (−0.03 to 0.50)^c^	0.076	−0.09 (−0.33 to 0.15)^c^	0.424	0.08 (−0.05 to 0.21)^c^	0.234^e^	−0.06 (−0.53 to 0.41)	0.789	0.03 (−0.12 to 0.18)	0.633	0 (−0.17 to 0.17)	1.000
9	0.37 (−0.02 to 0.75)	0.060	0.19 (−0.04 to 0.42)	0.097	0.12 (−0.02 to 0.26)	0.095	0.02 (−0.22 to 0.26)	0.881	0.08 (−0.06 to 0.23)	0.241	−0.01 (−0.28 to 0.27)	0.948	0.18 (−0.07 to 0.42)	0.109^e^	−0.01 (−0.13 to 0.11)	0.881	−0.07 (−0.24 to 0.09)^c^	0.332
10	−0.11 (−0.63 to 0.41)	0.650	0.10 (−0.14 to 0.34)	0.368	0.23 (−0.25 to 0.71)	0.315	0.24 (0.01 to 0.46)^c^	0.042	0.01 (−0.34 to 0.35)	0.954	0.02 (−0.39 to 0.43)	0.924	−0.13 (−0.28 to 0.03)	0.100	−0.05 (−0.34 to 0.23)	0.679	−0.05 (−0.25 to 0.14)	0.548

*Note:* Due to the multiple comparisons, statistical significance was set at *p* ≤ 0.01.

Abbreviations: CI, confidence interval; MD, mean difference; Min, minutes; MTPJ, metatarsophalangeal joint.

^a^ Significant difference between mean temperatures at comparative time points.

^b^ Significant difference likely attributed to Type I statistical error or statistical anomaly.

^c^ Extreme outlier identified and addressed by replacement with the next non‐outlier value.

^d^ Anatomical testing sites: 1, Plantar heel; 2, Lateral malleolus; 3, Medial malleolus; 4, Navicular tuberosity; 5, Dorsal midfoot (base of third metatarsal); 6, Cuboid; 7, Lateral fifth MTPJ; 8, Plantar third MTPJ; 9, Medial first MTPJ; 10, Plantar hallux.

^e^ Wilcoxon signed‐rank test was used when the distribution of the differences between time points was abnormally distributed (i.e., violated assumption for paired samples t‐test).

## 4 Discussion

This study is the first to explore the optimal time period to achieve temperature stabilisation when assessing dermal temperatures in active CNO. Our findings suggest that a resting period of 40 min is sufficient to acclimatise to ambient room conditions and to achieve a state of thermal equilibrium. These results advocate for re‐evaluating current practices and developing evidence‐based protocols for dermal temperature measurements.

Historically, healthcare professionals working within high‐risk foot services have practiced an acclimatisation period of 10–30 min post‐removal of offloading devices and contralateral footwear. A recent systematic review [4] found that a 15‐min resting time is most commonly reported [10, 14, 15, 16, 29, 30]. These acclimatisation (or resting) periods are largely based on methods from previous studies, general thermography guidelines, expert opinion and practical considerations, rather than high‐quality evidence [4, 10, 11, 14, 15, 16, 29, 30, 31]. Our findings challenge this convention, suggesting that a longer acclimatisation period of 40 min may be necessary to achieve optimal thermal equilibrium of the affected and non‐affected feet, particularly at anatomical sites with high clinical relevance (e.g., dorsal midfoot). Our data showed that the non‐casted foot had reached temperature stabilisation (i.e., no statistically significant differences between mean temperatures) by the 10‐min time point. The earlier stabilisation on the non‐affected foot likely correlates with the absence of a cast or offloading device providing insulative warmth to the skin. Not surprisingly, the casted foot took longer to stabilise, with all sites stabilising by the 40‐min time point. Notably, we observed statistically significant findings at certain anatomical sites and time points, including site 7 (lateral fifth MTPJ at 80 vs. 90 min) and site 9 (medial first MTPJ at 50 vs. 60 min) on the Charcot foot, as well as site 3 (medial malleolus at 50 vs. 60 min), site 5 (dorsal midfoot at 30 vs. 40 min) and site 7 (lateral fifth MTPJ at 30 vs. 40 min) on the non‐casted foot. However, given the absence of a clear trend preceding these statistically significant findings, these differences are likely attributable to Type I error or statistical anomalies, influenced by the combined effects of multiple comparisons (200 *t*‐tests in total) and the small sample size (*n* = 12), both of which increase the likelihood of Type I errors. Although these findings may not reflect true temperature variability, this remains uncertain and warrants further investigation. There may be merit in avoiding these sites when performing the dermal temperature assessment, but additional research is needed to confirm this and is beyond the scope of this study.

Accurate temperature measurement is critical for the early detection and management of CNO, as temperature differentials between the Charcot and contralateral foot can signal inflammation and potential complications [10, 15, 29, 31]. Indeed, it is emphasised in international guidelines [1] that a standardised approach to the dermal temperature measurement is essential to enable accurate comparison of repeated measures for monitoring progress and for clinical decision‐making. In addition, establishing standardised protocols may also help to facilitate meaningful comparisons across clinical and research settings [31]. Our study suggests that by establishing a 40‐min stabilisation period, clinicians could achieve more accurate and consistent temperature readings, thereby enhancing the diagnostic utility of infrared thermometry in this patient population. However, the feasibility of implementing a 40‐min resting period in routine clinical practice warrants consideration. Although this approach may enhance measurement accuracy, it may not be practical in all clinical settings due to time constraints and disruptions to patient flow. In busy clinical environments, such a prolonged stabilisation period might lead to inefficiencies and increased patient wait times. Therefore, future research should explore strategies to streamline the stabilisation process without compromising accuracy. Potential avenues may include the development of more rapid temperature equilibration techniques or the identification of specific foot regions that stabilise more quickly. Improving patient flow during clinical encounters could also be considered. Clinicians could prioritise tasks, such as removing the TCC and contralateral footwear and conducting any required diagnostic tests and treatments (e.g., x‐ray, neurovascular assessment and wound management), prior to performing dermal temperature measurements.

The duration of total contact casting has a significant impact on the individual and their overall health and wellbeing [18, 19]. As patients with active CNO are immobilised for long periods of time (median of 4.3 months [14]) and are required to reduce their weight‐bearing activity, there are often substantial and sustained negative impacts on patients' physical, social and emotional wellbeing [18, 19]. Limited mobility can adversely affect blood glucose control and cardiovascular health and can restrict social interactions and the ability to carry out work and/or family commitments [18, 19]. Driving is also an important consideration for patients, as it impacts employment, household income and the broader family dynamic in terms of caregiving responsibilities. The emotional and financial stress can vary between individuals depending on their previous commitments and employment. In a clinical setting, the temperature differential is often observed by clinicians to be a fixating point for the patient receiving care, and for many patients, it acts as a significant marker for improvement or deterioration in their condition. Therefore, ensuring that measurements are accurate and reliable is essential for effective and supportive patient care and for informing clinical decisions.

This study needs to be viewed considering some limitations. First, the footwear worn on the contralateral (non‐casted) foot was not standardised; differences in footwear can impact on skin temperature measurements, as certain materials and designs can insulate or expose the foot differently. Although this could have influenced our temperature measurements of this foot, the minimal number of statistically significant differences observed at the earlier time points suggest that this influence was likely negligible. In addition, only 3 of the 12 assessments were conducted during the summer months, with outside temperatures ranging from 11°C to 22°C and averaging 16°C, which is conducive to enclosed footwear. The practical necessity of wearing a supportive shoe to maintain even height when using a TCC also likely led to similar footwear choices for the contralateral foot among the participants. Second, ambient room temperatures were recorded only at the start of each data collection session rather than at 10‐min intervals or at the end of the session. Although more frequent measurements may have provided further insight into the potential influence of ambient temperature on dermal temperature readings, the likelihood of a significant impact is minimal. The average room temperature over the four seasons of data collection (June 2021 to March 2023) was 22.5°C with a standard deviation of only 0.4°C, indicating highly consistent environmental conditions. Additionally, despite external temperatures ranging from 11°C to 22°C during the study period, the facility maintained a stable indoor environment, demonstrating effective temperature regulation. Third, due to the small sample size (*n* = 12), there is an increased likelihood of the study being underpowered. As this was an exploratory study, there were no previous studies in this specific area to guide the estimation of an appropriate sample size or statistical power. Nonetheless, we enroled all eligible participants from our service, and recruitment was fully exhausted, meaning the maximum possible sample size was reached. Forth, due to the large number of *t*‐tests performed (100 tests per foot) and our small sample size (*n* = 12), there is an increased likelihood of Type I statistical errors, which seemed to be the case in the Charcot foot (sites 7 and 9) and the contralateral foot (sites 3, 5 and 7). To address this, the threshold for statistical significance was lowered to *p* ≤ 0.01. Fifth, our findings are mostly generalisable to people with diabetes‐related CNO (i.e., not from CNO related to other causes e.g., renal disease). However, given that diabetes is the leading cause of CNO, our results are representative of a significant portion of cases. Sixth, we conducted the dermal temperature assessments in a temperature‐controlled room. Although this is considered a methodological strength of our study, it may not accurately reflect the conditions in other clinical settings where these assessments are routinely performed. Finally, recall bias may have been a factor when participants were asked to self‐report the precipitant and duration of their Charcot foot. To mitigate this, we cross‐referenced the self‐reported data with medical records. There are several strengths of this study. To our knowledge, this is the first study to explore the optimal temperature stabilisation period for assessing dermal temperatures in active CNO. Our study had rigorous inclusion and exclusion criteria, study protocol and our findings are likely generalisable to clinical practice as our participants were recruited from a high‐risk foot service in a public health network, where the majority of CNO cases are managed.

Future research in dermal thermometry presents several promising opportunities to advance clinical practice and research methodologies. One avenue for exploration includes refining and standardising the methods for conducting dermal temperature measurements. This includes resting times, the optimal frequency of testing, testing techniques and assessing the variability across different anatomical sites. Understanding the interplay of these factors in diverse clinical contexts, such as varying environmental conditions or among different patient populations, is crucial for enhancing the applicability of dermal thermometry in healthcare settings. Innovations in technology could lead to the development of more precise and efficient temperature measurement devices, enhancing the accuracy and reliability of dermal thermometry in clinical settings. Additionally, investigating the correlation between dermal temperature patterns and disease progression or treatment outcomes could provide valuable insights into monitoring strategies and therapeutic efficacy. Furthermore, qualitative research into patient experiences and perceptions regarding dermal temperature assessments could help to inform clinicians on the psychological impact of this measurement, and in turn, how often these measurements should be performed.

## 5 Conclusions

This study provides valuable insights into the temperature stabilisation requirements for dermal thermometry in active CNO. A 40‐min resting period appears adequate for achieving thermal equilibrium in the Charcot foot and contralateral foot, potentially enhancing the diagnostic accuracy of temperature assessments. However, applying this finding in clinical practice poses challenges and highlights the need for continued research and innovation in this area. Our findings advocate for re‐evaluating current practices and developing evidence‐based protocols for dermal temperature measurements. A standardised approach is likely to improve the accuracy and reliability of temperature assessments, thereby enhancing the utility of these measures in clinical and research settings.

## Author Contributions

**Justin Bradley:** conceptualization, methodology, investigation, data curation, writing – original draft, writing – review and editing, visualization, project administration, funding acquisition. **Mollie Rumble:** conceptualization, methodology, investigation, data curation, writing – review and editing, project administration, funding acquisition. **Jennifer Wong:** conceptualization, methodology, writing – review and editing. **Ming Yii:** conceptualization, methodology, writing – review and editing. **Michelle R. Kaminski:** methodology, formal analysis, data curation, writing – original draft, writing – review and editing, visualization, supervision.

## Ethics Statement

This study was approved by the Monash Health Human Research and Ethics Committee (RES‐19‐0000‐794L). All participants provided written informed consent prior to enrolment and data collection.

## Consent

The authors have nothing to report.

## Conflicts of Interest

The authors declare no conflicts of interest.

## Supporting information

Table S1

## Data Availability

The data that support the findings of this study are available from the corresponding author upon reasonable request.
