# Supplementary material for: Optimal Time Period to Achieve Temperature Stabilisation After Total Contact Cast (TCC) Removal for Assessing Dermal Temperatures in Active Charcot Neuro‐Osteoarthropathy
Source: J Foot Ankle Res. 2025 Jul 25;18(3):e70059. doi: 10.1002/jfa2.70059 (PMC12461229; doi:10.1002/jfa2.70059)
Supplement: Supplementary file 1 — Table S1 [file JFA2-18-e70059-s001.pdf]

Appendix 1. Average temperatures (°C) at the 10 anatomical testing sites across 10 time points for the Charcot and contralateral foot – values are mean (SD)

**Charcot foot (casted)**										
**Site[a]**	**Baseline**	**10 min**	**20 min**	**30 min**	**40 min**	**50 min**	**60 min**	**70 min**	**80 min**	**90 min**
**1**	31.23 (1.58)	30.55 (1.44)	30.29 (1.68)	30.04 (1.84)	29.89 (1.79)	29.83 (2.18)	29.69 (2.07)	29.53 (2.11)	29.19 (2.06)	29.50 (2.22)
**2**	33.47 (0.62)	32.60 (0.89)	32.25 (0.99)	32.06 (1.13)	31.83 (1.11)	31.81 (1.20)	31.57 (1.51)	31.54 (1.51)	31.43 (1.46)	31.38 (1.53)
**3**	33.53 (0.80)	32.70 (0.68)	32.35 (0.86)	32.21 (0.97)	32.09 (0.85)	32.06 (0.95)	31.91 (1.03)	31.83 (1.03)	31.78 (1.16)	31.76 (1.13)
**4**	33.27 (1.00)	32.54 (0.69)	32.21 (0.86)	31.98 (1.04)	32.00 (0.91)	32.00 (0.92)	31.79 (1.12)	31.64 (1.10)	31.67 (1.25)	31.57 (1.27)
**5**	33.59 (0.99)	33.17 (1.06)	32.98 (1.14)	32.74 (1.24)	32.61 (1.21)	32.48 (1.27)	32.37 (1.38)	32.27 (1.47)	32.16 (1.50)	32.07 (1.55)
**6**	33.23 (0.92)	32.26 (0.98)	31.73 (1.16)	31.52 (1.31)	31.36 (1.26)	31.20 (1.48)	31.07 (1.48)	30.83 (1.49)	30.87 (1.48)	30.74 (1.60)
**7**	31.18 (1.55)	30.36 (1.67)	29.74 (1.78)	29.76 (1.94)	29.44 (2.31)	29.48 (2.30)	29.32 (2.19)	29.15 (2.20)	29.29 (2.22)	29.01 (2.29)
**8**	31.83 (1.22)	31.25 (1.41)	31.03 (1.46)	30.88 (1.55)	30.72 (1.69)	30.53 (1.60)	30.46 (1.67)	30.36 (1.69)	30.19 (1.83)	30.13 (2.04)
**9**	32.07 (1.65)	31.18 (1.57)	31.06 (1.66)	30.63 (1.80)	30.41 (1.80)	30.42 (2.02)	29.98 (2.17)	30.01 (1.95)	29.92 (2.07)	29.79 (2.21)
**10**	29.85 (2.22)	29.35 (2.48)	29.29 (2.37)	28.91 (2.63)	28.66 (2.54)	28.66 (2.61)	28.50 (2.70)	28.33 (2.73)	28.19 (2.69)	28.10 (2.67)
**Contralateral foot (non-casted)**										
**Site[a]**	**Baseline**	**10 min**	**20 min**	**30 min**	**40 min**	**50 min**	**60 min**	**70 min**	**80 min**	**90 min**
**1**	29.10 (2.07)	28.75 (2.11)	28.48 (2.10)	28.37 (2.23)	28.09 (2.04)	28.13 (2.19)	28.13 (2.29)	28.03 (2.33)	27.91 (2.38)	27.81 (2.54)
**2**	29.47 (2.23)	29.35 (2.06)	29.38 (2.02)	29.18 (1.89)	29.14 (1.91)	28.98 (1.88)	28.94 (1.68)	28.98 (1.82)	28.96 (1.92)	28.93 (2.02)
**3**	30.60 (2.08)	30.48 (1.74)	30.43 (1.72)	30.24 (1.65)	30.17 (1.66)	30.13 (1.64)	29.95 (1.63)	29.81 (1.63)	29.93 (1.72)	29.76 (1.72)
**4**	30.17 (2.19)	29.74 (2.12)	29.64 (2.03)	29.46 (1.94)	29.28 (1.88)	29.30 (1.90)	29.28 (1.85)	29.16 (1.79)	28.97 (1.85)	28.87 (1.82)
**5**	30.38 (2.69)	30.19 (2.55)	30.11 (2.59)	29.93 (2.38)	29.68 (2.31)	29.78 (2.24)	29.68 (2.19)	29.60 (2.21)	29.62 (2.24)	29.46 (2.31)
**6**	29.42 (2.45)	29.05 (2.18)	28.87 (2.07)	28.70 (1.99)	28.52 (1.85)	28.44 (1.96)	28.32 (1.76)	28.23 (1.88)	28.38 (2.01)	28.23 (2.00)
**7**	27.60 (3.22)	27.48 (2.93)	27.48 (3.10)	27.33 (3.01)	27.02 (3.01)	27.17 (3.11)	27.18 (3.14)	27.07 (3.13)	26.97 (3.00)	26.91 (3.05)
**8**	28.24 (3.06)	28.21 (3.25)	28.25 (3.29)	27.99 (3.18)	27.76 (3.20)	27.85 (3.20)	27.77 (3.17)	27.83 (3.38)	27.79 (3.38)	27.79 (3.36)
**9**	27.79 (2.63)	27.43 (2.76)	27.23 (2.73)	27.12 (2.64)	27.10 (2.74)	27.02 (2.60)	27.03 (2.61)	26.85 (2.44)	26.86 (2.56)	26.93 (2.56)
**10**	26.18 (2.42)	26.29 (2.78)	26.19 (2.78)	25.96 (2.42)	25.73 (2.46)	25.72 (2.69)	25.70 (2.95)	25.83 (2.85)	25.88 (2.76)	25.94 (2.84)

Min, minutes; MTPJ, metatarsophalangeal joint; SD, standard deviation.

[a]Anatomical testing sites: 1, Plantar heel; 2, Lateral malleolus; 3, Medial malleolus; 4, Navicular tuberosity; 5, Dorsal midfoot (base of 3rd metatarsal); 6, Cuboid; 7, Lateral 5th MTPJ; 8, Plantar 3rd MTPJ; 9, Medial 1st MTPJ; 10, Plantar hallux.
